# Process evaluation of the randomized trial—“Care management for older people with cognitive impairment during and after hospitalisation in Germany (intersec-CM)”

**DOI:** 10.1186/s13063-026-09965-0

**Published:** 2026-08-29

**Authors:** N. Chikhradze, A. Hannich, IC. Otte, T. S. Busse, A. Suslow, A. Nikelski, F. Schumacher-Schoenert, S. H. Kreisel, M. Boekholt, J. R. Thyrian, W. Hoffmann, H. C. Vollmar

**Affiliations:** 1 Medical Faculty, Institute of General Practice and Family Medicine (AM RUB), Ruhr University Bochum (RUB), Universitätsstr. 150, Bochum, 44801 Germany; 2 Medical Faculty, Institute for Diversity Medicine, Ruhr University Bochum (RUB), Universitätsstraße 105, Bochum, 44789 Germany; 3 Institute for Community Medicine, University Medicine Greifswald, Ellernholzstr. 1/2, Greifswald, 17489 Germany; 4 Junior Professorship for Digital Health, Department of Human Medicine, Faculty of Health, Witten/Herdecke University, Alfred-Herrhausen-Str. 50, Witten, 58455 Germany; 5 Division of Geriatric Psychiatry, Medical School and University Medical Center OWL, Protestant Hospital of the Bethel Foundation, Bielefel, Bethesdaweg 12, Bielefeld, 33617 Germany; 6 German Centre for Neurodegenerative Diseases (DZNE), Site Rostock/Greifswald, Ellernholzstr. 1-2, Greifswald, 17489 Germany

**Keywords:** Process evaluation, Cognitive impairment, Intersectoral care management, Intersec-CM

## Abstract

**Background:**

Effective care of people with cognitive impairment and their families depends, among other things, on the cooperation of the professionals involved in their care. An effective dementia care management support model for people with dementia and their families for the outpatient sector has been further developed for ambulatory and hospital settings. The intersec-CM randomized controlled trial (RCT) implemented this intersectoral care management (iCM) model into routine care in three hospitals in Bielefeld and Greifswald (Germany). Care managers (CMs) trained in advance for the study used a computer-based information management system (IMS) to assess patients’ health and care needs and to develop individualized care plans. The iCM aimed to improve hospital-to-primary-care transitions and ensure cross-sectoral care. This was accompanied by a process evaluation to complement the data collected in the RCT. The aim was to understand how the complex intervention works in the selected real-world setting. This paper presents the results of this process evaluation.

**Methods:**

For the process evaluation, a mixed-method design was chosen and applied. The evaluation was based on the guidelines of the British Medical Research Council for the implementation and process evaluation of complex interventions (MRC framework). A method triangulation was conducted consisting of several successive phases (t1: qualitative, t2: quantitative, t3: qualitative).

**Results:**

The sample (t1: individual interviews *n* = 30, t2: survey *n* = 177, t3: individual interviews *n* = 33) consisted of patients and their relatives, clinical physicians, social workers, carers, general practitioners, care managers (CMs), and project management. The process evaluation showed that intersectoral iCM could be implemented in the hospitals. The CMs were partially successful in assessing needs, developing individual treatment plans, and coordinating and monitoring the needs of people with cognitive impairment and their families. They found it difficult to work with the different actors in the healthcare system because of the rigid daily routines and lack of time in the clinics. Overall, the results show that (1) people with cognitive impairment and their families can benefit from the intervention and (2) iCM can help with the flow of information between health professionals.

**Conclusions:**

The present process evaluation underlines the necessity and meaningfulness of process evaluations to understand the functionality and procedural feasibility of an RCT. Through the study, significant insights into the “black box” of the implementation of the iCM intervention could be gained.

**Trial registration:**

ClinicalTrials.gov NCT03359408. The trial was registered on December 2, 2017.

**Supplementary Information:**

The online version contains supplementary material available at https://doi.org/10.1186/s13063-026-09965-0.

## Background

It is known that people with cognitive impairments and their relatives represent a vulnerable group [1–6]. The successful care for these people depends, among other things, on the cooperation of the professional groups involved in their care [7]. This is particularly relevant when care is not provided in the same facility and across sectors. While in Germany, the health care system is structured into distinct sectors: (1) outpatient treatment and care, (2) inpatient treatment and care, and (3) rehabilitation [8], special tasks are required here. Many people transition between these sectors in their continuum of care, which requires diligence regarding the communication between the various healthcare providers involved to share care needs and plans without information loss [9, 10]. However, it is not only the exchange between care providers but also the extended environment that is relevant for the quality of care—patient and family involvement is positively associated with the health behavior and treatment outcomes of patients [11].

Especially in Germany, there is therefore a need for a sustainable concept that encompasses treatment and care and is geared more to the needs of patients and their relatives than to the needs of hospitals or specific diseases [5, 12, 13]. Recent specifications on discharge management in Germany intensified this need [10–14, 14, 15]. To date, there have been only a few studies in this area, but those that have been conducted have provided extensive evidence: Results from DelpHi-MV (Dementia: Life- and person-centred help in Mecklenburg-Western Pomerania, Germany (DelpHi)) study of a dementia care management (DCM) model for the outpatient sector showed that the model was an effective support for people with dementia, family members, and general practitioners (GPs). The study showed that close cooperation and communication between the different professional groups was an important success factor of the intervention [15]. The DeCM concept was further developed for hospitals and implemented into routine care in the clinics in Bielefeld and Greifswald in the randomized controlled study (RCT) intersec-CM [3, 10, 16]. Thus, the intersectoral care management (iCM) was used in this study to improve patient-oriented treatment and care in the transition from the hospital to primary care, as well as to ensure cross-sectoral care for these people [2, 3].

### Description of the intersec-CM intervention (iCM)

Within the iCM intervention, Care managers (CMs) trained in advance for the study used a computer-based information management system (IMS) on tablets to comprehensively assess the health and care situation of participants and to develop an individualized care plan [3, 16]. Towards the end of the hospital stay, the CMs were to discuss the plan with the patients and their relatives and accompany and coordinate its implementation at home. The intention of the intervention was to accompany the discharge management in a patient-oriented way and to involve relatives in this process. In addition, CMs should work in both inpatient and outpatient settings with all professionals involved in care to optimize the flow of information between distinct health sectors. In doing so, the CMs should not replace the activities of other professional groups, such as hospital physicians, nurses, social workers, and GPs (ambulatory) as well as specialist physicians (ambulatory) [3], but support all of these involved in the care of a patient in this study.

The RCT in the intersec-CM trial [3, 10, 16] had the aim to evaluate the effectiveness of the intervention as a complex intervention [17]. However, to improve care and apply the findings from the intersec-CM RCT, it is relevant to understand how it was implemented in its specific context, how different healthcare professionals were involved, and whether this intervention can be applied to routine care. Therefore, a process evaluation was conducted with the aim of supplementing the data collected in the RCT and gaining an understanding of how and to what extent this complex intervention was implemented under routine conditions. This aligns with the fact that the relevance and importance of process evaluations are increasingly recognized in scientific discourse and represent an important quality criterion [17–21].

Process evaluations are strongly recommended for complex interventions. Intersec-CM can be classified as a complex intervention since it meets the following criteria: (1) multiple interacting components, (2) high difficulty of implementation, and (3) involvement of multiple organizational levels [22]. It is known that, in addition to the practical and methodological difficulties, a number of specific challenges arise when implementing complex interventions in healthcare. These are often due to the different dimensions of the complexity of the intervention and to the constantly changing routines in the system. As interventions are affected by these changes [19, 23], the interacting components need to be explored in the process evaluation [24]. For this reason, based on the aim of this process evaluation and on the guidelines of the British Medical Research Council for the implementation and process evaluation of complex interventions (MRC framework), this study addresses the following questions regarding different dimensions of the MRC framework:

- Under which contextual conditions was the intervention implemented?—MRC dimension: context
- Which implementation steps were taken?—MRC dimension: implementation
- How was the intervention delivered and did the intervention change?—MRC dimension: implementation
- What impact factors can be identified?—MRC dimension: implementation

### Causal assumptions on intersec-CM

Accordingly, it was necessary to explore multiple interacting components within intersec-CM: the difficulty of the behaviors required for those delivering or receiving the intervention; the groups or levels of the organization targeted by the intervention; and the degree of flexibility of the intervention [19]. In this way, the process evaluation can assess the reliability and quality of the implementation of iCM as a complex intervention and uncover mechanisms of the observed effect as well as identify contextual factors. In addition, it can shed light on what was done differently in the intervention, or at what point in the implementation process difficulties and barriers [25].

As an integral part of the randomized controlled intersec-CM trial, the results of a process evaluation are presented in this paper [17]. Against this background, this paper maps the implementation of care management under routine care conditions in the intersec-CM study.

## Methods

For the process evaluation, a mixed-methods design was chosen and applied [19, 26–30] by three researchers (two female and one male) with expertise in qualitative, quantitative, and mixed-methods approaches. The reporting of this study is aligned to the COREQ guideline [31] (see Appendix 1).

**Fig. 1:**
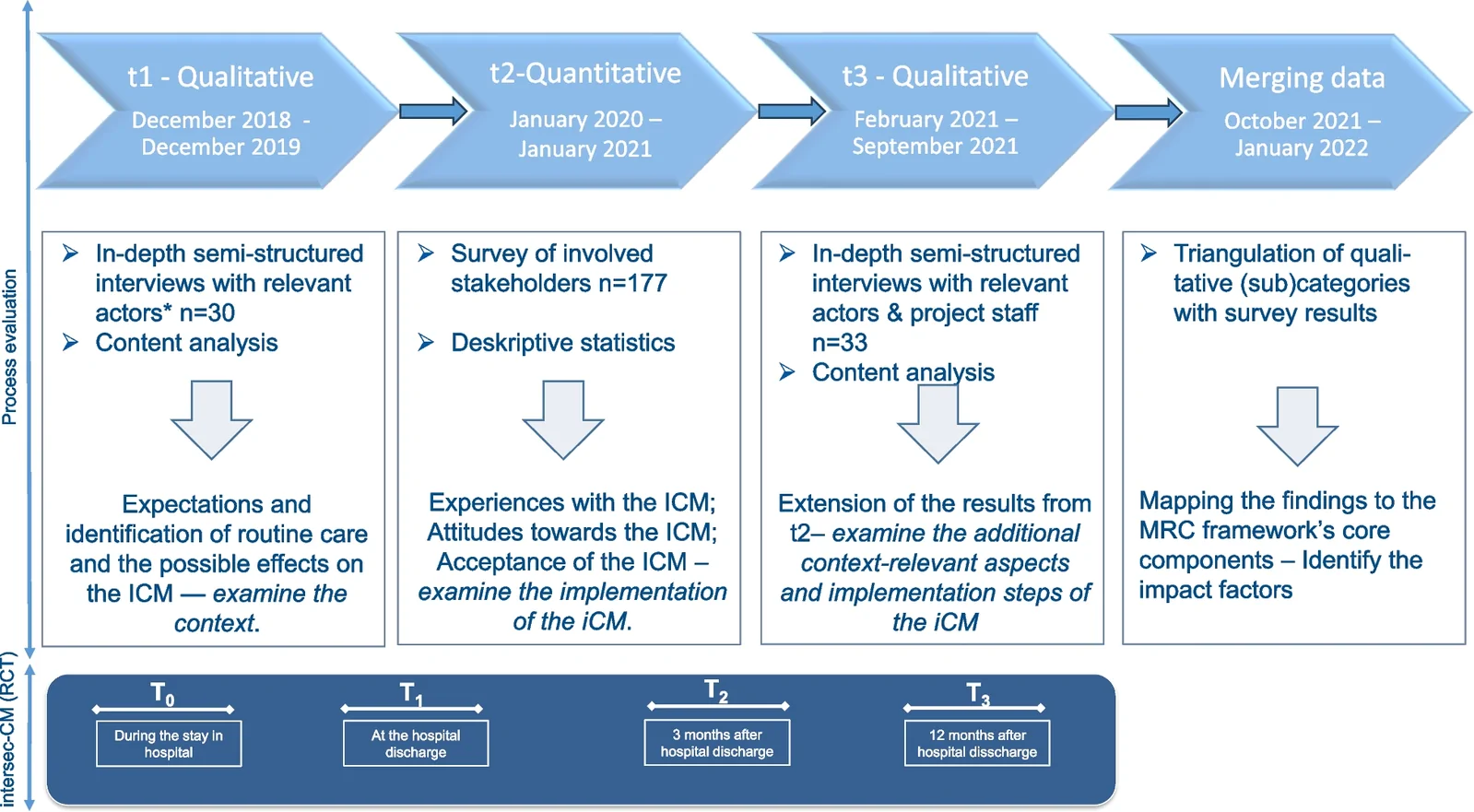
Study design and overview of sequential study phases

As recommended by Prick et al. [32], a method triangulation consisting of several successive phases (see Fig. 1) was conducted (t1: qualitative, t2: quantitative, t3: qualitative) [26, 33, 34].

The evaluation and its presentation were based on the guidelines of the British Medical Research Council for the implementation and process evaluation of complex interventions (MRC framework). This recommends the recording of different factors that influence the results and provide an insight into the implementation process [20]. The model maps the key functions of the process evaluation and their interrelationships. Therefore, the MRC Guidance was embedded in the process from the first methodological step to the end of the data analysis. The primary objective of the initial (t1) data collection was to examine context-relevant aspects of the setting. Therefore, the interview guides included themes related to general knowledge and prior assumptions regarding admission to, treatment in, and discharge from the hospital. During data collection at t2 and t3, the focus was on acceptance as an intention to adopt the iCM (as a key factor for the successful implementation of interventions in healthcare) and on identifying how and to what extent the iCM was implemented. Accordingly, these interviews included questions about acceptance, which were directed at users (i.e., healthcare professionals) and target groups (i.e., patients and relatives). They were asked about training for the intervention, whether they considered iCM was suitable, meaningful, and practical, how they collaborated with Care managers (CMs) to ensure older people were discharged from hospital in line with the intervention, and what their wishes were for the future.

The procedure of the process evaluation in the intersec-CM study was defined in a study protocol [30].

### Participants of the process evaluation

Participants from different groups were recruited for the various data collections (t1, t2, t3) in order to ensure a diversity of perspectives. The inclusion criteria for the participants of the process evaluation were evolved based on the Actor-Network Theory (ANT) of sociologist Bruno Latour [35]. Accordingly, the process evaluation at t1 and t2 included older people with cognitive impairments and their relatives as well as healthcare professionals including nurses, social workers, clinicians, GPs, specialist practitioners, and CMs [30].

While the data collection at t3 aimed to better understand the processes within the RCT and identify reasons for variation in implementation success [29], project management and scientific staff were interviewed about their experiences within the project. Additionally, nurse experts from the clinical area who were not involved in the RCT were recruited to be interviewed about their attitudes toward the iCM intervention, to better understand the structural aspects that hindered collaboration between the CMs and hospital staff, and to identify how this collaboration could be optimized for discharge processes in their hospitals. Also included in the t3 data collection were relatives of people with cognitive impairment who had experience with hospital discharge of relatives but had no experience with care provided by a CM. It was essential to verify that the survey results (t2) demonstrating the benefits to patients and their families were not limited to a specific subgroup. Including diverse relatives therefore strengthened the credibility of the findings. Once all data analyses were completed, a focus group discussion was conducted with the RCT project management and scientific staff to address any remaining questions.

### Data collection procedure t1, t2, t3

For the first phase of data collection (t1), semi-structured interview guides were developed, based on the available data from the reviewed international literature on routine inpatient care of people with cognitive impairment [36]. The aim was to collect the experiences and wishes of the relevant participants during hospitalization, treatment, and discharge from hospital prior to the implementation of the intervention with the focus to examine the contextual factors of the setting as recommended in MRC Guidance. For this purpose, further needs regarding the care of patients with cognitive impairments and hospital specifics were recorded. The interviews (t1) were conducted between December 2018 and July 2019 by the first author of the present publication who holds a doctoral degree and a researcher with a master’s degree (C.N., L.P.). Because the implementation of the intervention in the participating hospitals began in November 2018, interview partners who had not had contact with the intervention were selected by staff at these hospitals.

Based on the results of the semi-structured interviews (t1), a quantitative survey (t2) was developed by two researchers (C.N., L.P.) and discussed within the research team to interview the stakeholders between 2019 and 2020 (t2). The surveys at t2 were sent to the participants with a pre-stamped return postage envelope and the study documents. The study documents consisted of a cover letter containing a brief description of the study intervention, a consent form, and a request to make themselves available as potential interview partners for the following qualitative data collection (t3). People who did not respond by mail received telephone calls as reminders. Additionally, participants were informed about potential biases, assumptions, reasons, and interests related to the research topic. In the third phase of the evaluation in 2021 (t3), semi-structured guideline interviews were conducted by the authors of the present publication (C.N., H.A.) to gain a deeper insight into the project experiences and to record the wishes of the people involved in discharge management. The guidelines used for the interviews were developed based on the findings of the previous data collection phases (t1, t2) by two researchers and were also discussed in the interdisciplinary research team.

The t1 and t2 interviews were conducted in person and via telephone/online in the context of infection control during the COVID-19 pandemic by researchers of the respective site involved in the process evaluation.

The guided interviews in phases 1 and 3 were voice recorded and transcribed according to the extended rules of Dresing and Pehl [37].

### Data analysis

The qualitative content analysis according to Kuckartz [36] was chosen to explore the complex processes, experiences, and perspectives of the iCM implementation and data were analyzed with predefined coding rules using MAXQDA 2020 [38]. The analysis of the interviews was carried out by two members from various disciplines such as nursing science (1) and health sciences (2) of the study team using a consensual coding procedure. First, the overarching categories were formed deductively based on the research question and the MRC framework. These included, among other things, contextual factors within hospitals, as well as implementation features such as training and initial patient contact and relatives by case management (CM), care processes from first contact to discharge, and the initial home visit to patients, among others.

The initial data from three interviews were independently and line-by-line open-coded by two researchers to identify relevant text segments. Subsequently, coding trees with (sub)categories were developed. These coding trees were discussed between the researchers and merged into a consensus-based coding tree.

This (sub-)coding tree was divided into the overarching categories that were developed deductively. Further analyses of the data were then conducted based on this framework to identify additional categories and analyze the data as a whole. Categories and subcategories were further refined through the involvement of the peer group consisted of scientists from various disciplines such as medicine (1), nursing science (2), and health sciences (2) in the monthly RCT meetings. Based on the initial qualitative results, the survey items were developed to either confirm or refute them. The quantitative data were analyzed separately by two researchers, and any questions that arose from the results were incorporated into the interview guide to expand the scope of the findings. In a further step, the categories and subcategories that were created were compared with the results of the quantitative survey and with the key components of the MRC framework and assigned to them [20].

A central theme of this process evaluation is that several contextual factors hindered the implementation of the intervention. Internal hospital-related factors included insufficient daily structure and the need for patient referrals to GPs. External factors, related to the RCT and the broader health system, included insufficient technical equipment, high staff turnover, the pandemic situation, the dual role of the care managers (CMs), and a flat hierarchy within the project.

During the implementation phase, four key themes were identified:

- Onboarding: CMs and hospital staff gained knowledge about the project and the intervention.
- Pilot/adaptation phase: The originally planned goal of close CM-GP contact was removed from the intervention.
- In hospitals: CMs often needed to identify “gaps in daily clinical practice” to perform their tasks, which impeded systematic execution of the intervention.
- Outpatient care: CM activities mainly focused on providing emotional and mental support to patients.

This structure mirrors the workflow from contextual factors to implementation outcomes as shown in Fig. 5.

The quantitative data of t2 were analyzed descriptively using SPSS 28 (IPM, New York), which indicated challenges related to the implementation of the intervention, particularly regarding insufficient collaboration between CMs and hospital staff. In addition, the results suggested that hospital staff had limited awareness of the intervention within their daily clinical practice. Based on these findings, specific aspects were further explored and refined during the t3 data collection. Data were triangulated in the subsequent step. The qualitative component from t2 allowed for a more in-depth examination of the issues, providing contextual insights into barriers to collaboration, communication gaps, and the perceived integration of the intervention into routine care. By combining quantitative trends with qualitative explanations, the analysis enabled a more comprehensive understanding of the implementation process and its challenges.

## Results

The presentation of the results of the process evaluation starts with the description of the study sample. Following this, results oriented toward the MRC framework [20], beginning with the contextual factors, are presented in the Context section. In the following Implementation and What is delivered sections focusing on implementation, the process of application as well as the fidelity, dose, adaptations, and reach are described using the data from t1, t2 and t3. Subsequently, what is delivered with the impact factors is described.

### Study sample

The sample consisted of participants from three hospitals at the two study sites at Bielefeld (*n* = 1) and Greifswald (*n* = 2).

The sample for the t1 qualitative interviews comprised 15 participants per site. The age ranged from 29 to 86 years. The marital status, the presence of children, level of education, and employment were collected for the exact description of the participants. Table 1 provides an overview of the whole sample in Bielefeld and Greifswald.

**Table 1:** Sample t1 and t3; * not involved in the study

Sample/location	Greifswald		Bielefeld		Total	
	t1	t3	t1	t3	t1	t3
Participants/*N*	*N* = 15	*N* = 18	*N* = 15	*N* = 15	*N* = 30	*N* = 33
Care managers (w/m)	2 (2/0)	2 (2/0)	2 (2/0)	2 (2/0)	4 (4/0)	4 (4/0)
Project management and scientific staff	–	2 (1/1)	–	1 (1/0)	–	3 (2/1)
Persons with cognitive impairment (w/m)	2 (1/1)	2 (1/1)	2 (2/0)	3 (2/1)	4 (3/1)	4 (3/1)
Relative (w/m)	2 (1/1)	2 (2/0)	2 (2/0)	2 (1/1)	4 (3/1)	4 (3/1)
Relatives as experts (w/m)	–	2 (2/0)	–	2 (2/0)	–	4 (4/0)
Clinical nurses (w/m)	3 (3/0)	3 (3/0)*	2 (1/1)	3 (1/2)*	5 (4/1)	7 (5/2)*
Hospital social workers (w/m)	2 (2/0)	2 (2/0)	2 (2/0)	0 (0/0)	4 (4/0)	2 (2/0)
Hospital physicians* (w/m)	2 (0/2)	1 (1/0)	2 (1/1)	0 (0/0)	4 (1/3)	2 (2/0)
General practitioners* (w/m)	2 (1/1)	2 (1/1)	3 (2/1)	2 (1/1)	5 (3/2)	4 (2/2)
Participants’ demographics						
Family members in need of care: yes/no	3/15	4/14	2/13	5/10	5/28	9/24
Age (years): median	53	56	50	62	51.5	59
Marital status						
Single	2	2	3	0	5	2
Live in partnership/married	9	13	10	12	19	25
Widowed	4	3	2	3	6	6
Children: yes/no	10/5	17/1	11/4	14/1	21/9	31/2
Education						
< High school	6	6	3	5	9	11
High school	3	12	5	8	8	20
Some college	6	0	7	2	13	2
Socio-professional category	13	13	13	8	26	21
Worker	0	0	0	1	0	1
Housewife/houseman	2	5	2	6	4	11
Retiree	0	0	0	0	0	0
Seeking work	0	0	0	0	0	0
Other	0	0	0	0	0	0

For the t2 survey, a low initial response rate (3%) became apparent at both locations. After multiple telephone contacts (2–5 times per respondent), *n* = 177 questionnaires were completed of the *n* = 434 questionnaires sent out (Table 2).

**Table 2:** Sample t2

Participants	Total *N* (%)	Location		Gender		Age, years, median
		Bielefeld *N* (%)	Greifswald *N* (%)	Male *N* (%)	Female *N* (%)	
Patients	89 (50)	37 (42)	52 (58)	29 (33)	60 (67)	83.3 (5.4)
Relatives	14 (8)	11 (79)	3 (21)	1 (7)	13 (93)	76.6 (10.4)
General practitioners	46 (26)	10 (22)	36 (78)	24 (52)	22 (48)	53 (9.3)
Hospital physicians	2 (2)	1 (33)	2 (67)	3 (100)	0 (0)	40 (11.3)
Clinical nurses	15 (8)	7 (47)	8 (53)	4 (27)	11 (73)	41.8 (10.8)
Hospital social workers	7 (4)	5 (71)	2 (29)	1 (14)	6 (86)	37.0 (15.4)
Care managers	3 (2)	1 (33)	2 (67)	0 (0)	3 (100)	/
Total	177 (100)	72 (41)	105 (59)	65 (37)	112 (63)	

At t3, 15 participants were interviewed in Bielefeld and 18 participants in Greifswald. The items of the t3 sample are the same as at t1. A more detailed list of the characteristics represented by the participants can be found in Table 1.

### Context

Contextual factors can be divided into internal hospital-related and external, RCT- and general health system-related factors coming up in the RCT as well as general health system-related factors. Regarding the hospital-related factors, it was noticeable that the hospitals at both study sites had multidisciplinary centers with several clinics. Their discharge management was carried out by the hospital’s social services.

#### Internal hospital-related factors

##### Insufficient daily structure

People working in the hospitals’ social services have to deal with various challenges in the context of discharge management. One hospital-related factor was the rigid and clocked daily structure that leaves little time for planning discharge management with the patients:

... to always have to interfere in this everyday life and to meander through in order to really come into contact with the patients and also to be able to conduct an assessment that was always very challenging. (Care manager, t3)

Such a daily structure of the hospital also influenced the sensitive conversations that Care managers (CMs) were having with patients and families. Additionally, when there was time to talk to patients and their relatives about discharge management, one hospital-related factor was the limited privacy in the ward rooms. CMs reported that conversation was often interrupted by the daily routine activities on the ward. The CMs described that it was not possible to talk in private because other patients were in the room or visitors were present.

##### Referral to GPs

One of the contextual factors affecting implementation is the referral to relevant GPs in the discharge management, which is desirable for seamless support. Participants in interviews complained about the lack of availability of GPs for hospital staff and in general about the lack of dialogue between hospitals and the outpatient sector. This creates a gap in the network which, according to a social services worker, has led to difficulties in organizing the transition from hospital to home.

So maybe the problems are the GPs. I think so because our doctors don’t communicate with the GPs like that. We can’t get close to the GPs, who are sometimes unwilling to do anything. (Social worker, t1)

#### External, RCT- and general health system-related factors

##### Insufficient technical equipment

The external contextual factors include the technical equipment that was available for the implementation of the project at both sites. The data implies that the IMS was perceived as not flexible enough for the preparation of the care plans and did not cover certain needs. The CMs reported that it was not possible to record the necessary information, such as a patient’s social situation, information from discharge letters, prescribed medications, and aids, in the tablet in order to develop a personalized treatment plan. As a result, CMs wrote down the necessary information separately by hand, in addition to the tablets, to enable them to provide care to patients and their families.

(…) And then we wrote down the social situation, wrote down the medication, wrote down the diagnoses, copied discharge letters into the case file, all sorts of other important documentation from our hospital information system, I don’t know when they went home, what aids they ordered from social services, things like that. I couldn’t have documented that anywhere in the tablet. (Care manager, t3)

There were also some functional problems with the tablets for the IMS, such as the lack of compatibility of the software with the outdated technical features of the tablets. This made it difficult to implement the intervention on a daily basis; when using the tablets, the system crashed and patient data had to be re-entered:

(...) yes, so for me the biggest problem was that this IMS is still not so mature, that there, so that it can never be perfect, but that it, that it was further worked on or that it was intensified, that was already very (...) almost said larifari, (...) (Care manager, t3)

##### High staff turnover

Employee turnover is another one of the external factors. A high turnover of staff in the hospital was reported as affecting the cooperation between project team and clinic staff. Similarly, the implementation of the intervention at both sites was influenced by the staff changes during the RCT and, according to one project leader, made the implementation of the project more difficult:

So this is (laughs) one of my projects where the staff turnover was the biggest (laughs) so it was quite bad, staff turnover, in the planning of the project there were others who were in the implementation and then again others who are now in the further implementation, a huge problem. (Project manager, t3)

As a result, key contacts on the wards were often no longer available, and the study content had to be explained again to new contacts:

(...) another problem we had was the EXTREME staff turnover in the hospital, so that contacts were changing like crazy, and in some cases the processes were not clearly defined for their normal routine work at all... (Project manager, t3)

Staff turnover among the CMs was also notable. At one site, a CM reported in a T3 interview that she had missed much of the hospital phase of the RCT because she had joined the project at a later stage. She also emphasized that this had made establishing contact more difficult, explaining:

So, perhaps it was a bit strange for people to confide in someone who was completely new, but I think for many it didn’t play such a big role. For some, yes, but for others, not so much (Care manager t3)

##### Pandemic situation

A major external factor that had a significant impact on the cooperation between the participants and the CMs and thus influenced the implementation of the intervention was the COVID-19 pandemic. One consequence was that contact between the CMs and the patients or their relatives was only possible by telephone.

(...) because of the coronavirus, the home visits were cancelled afterwards, and it is simply difficult to cover everything in a telephone survey. (Care manager t3)

In several cases, phone calls were further complicated because older adults either feared being deceived in so-called grandparent scam calls or were no longer familiar with using the telephone. This is consistent with the t2 results, which showed that 21 of the 89 participants had not noticed the project (Fig. 4). As a result, continued contact with several participants was not possible, and aspects of the intervention could not be fully delivered:

And yes, I think those who really were no longer good with the phone just didn’t pick up. There were also many people with whom it was impossible to establish any contact at all. (Care manager, t3)

##### Dual role of the CMs

From the CMs’ perspective, the project posed a number of challenges. One of the internal hospital-related factors during the implementation of the project was the dual role of the CMs that in some instances caused conflicts between CMs tasks: On the one hand, the CMs carried out the practical tasks of the interventions; on the other hand, they recorded the effectiveness of the study through assessments. This resulted in a conflict between practical and scientific tasks for them:

This is a very difficult role because they have this double role. On the one hand, they are a case manager or care manager and are responsible for the immediate needs of the patients, and they also feel responsible. And on the other hand, they also have the role of a study assistant. And that is, I think, difficult, to change hats more often. (Project manager, t3)

##### Flat hierarchy within the project

The implementation was characterized by a flat hierarchy within the project. The cooperation between the CMs, project and consortium managers was described as constructive and solution-oriented by the participants. In case of problems, solutions and possibilities for implementation were sought cooperatively. The implementation was accompanied on site in Bielefeld by the project’s scientific manager. To ensure implementation on the part of the CMs, weekly case conferences were held between the study management and the study site on the one hand, and between the project staff within the study site for monitoring and quality assurance on the other. Since the project stuff was working on the same premises, it was possible to work closely together. This resulted in spontaneous discussions about problems and, when necessary, the initiation of supervision.

In Greifswald, the CMs reported that they did not need this form of support because they were already familiar with the IMS and the intervention through the previous DelpHi-MV study [15, 38, 39]. Therefore, they exchanged information with each other on a day-to-day basis and acted largely independently. The project management trusted the approach of the CMs in implementing the interventions:

(...) so like our care manager XY, she didn’t have to do a lot of job shadowing, she has been doing it for years (...) (Project manager, t3)

### Implementation

The actions and processes of implementing iCM within the project can be divided into the following phases (see Fig. 5):

- Onboarding: gain knowledge through the project.
- Pilot phase/adaptation of the intervention: the goal of close contact through CM-GP, removed from the intervention.
- In hospitals: look for gaps in the daily routine to take action as CM.
- Outpatient care: emotional and mental support from CMs.

The following describes the phases and the results regarding the hurdles and supporting factors identified in each phase through the process evaluation.

#### Onboarding: gain knowledge through the project

The intersec-CM study started in March 2018 with a 3-day inter-site project meeting of the staff of the participating departments at the three hospitals. The aim of this meeting was to get to know the staff and the structures, to network the staff of the locations, to exchange ideas and to explain the intersec-CM project procedure. Relevant content of the assessments and their timing within the study was also communicated:

We met once and looked at some assessment instruments for half a day. (Project manager, t3)

CMs taking part in the study had attended training on topics such as geriatric psychiatry, hospital delirium prevention program, family care, physician network/case management, IMS, communication/caring for people with cognitive impairment, delirium, and frailty syndromes, data protection and the duty of confidentiality, discharge management, and challenges in care (conflicts, aggression, and violence).

The preparation phase continued with a presentation of the study to the stakeholders in the participating clinics. The Care managers (CMs) were introduced to the relevant hospital staff on duty on the wards and the intervention contents were discussed with the staff involved in discharge management. A special focus was placed on the timing and processes of data collection. This was to prepare and promote cooperation between CMs and clinic staff in advance. In parallel, the study was described on the websites of the respective clinics and relevant contact persons were identified. Thus, clinic staff and external visitors of the websites had the opportunity to read up on the specific study contents independently and to contact the study team directly in case of open questions.

#### Pilot phase/adaptation of the intervention: the goal of close contact through CM-GP, removed from the intervention

The next step in the implementation process was to conduct a three-month pilot phase. During this time, the study staff were able to observe the implementation under realistic practice conditions. It turned out that both the implementation process and the intervention contents needed to be adapted to the actual conditions in the respective clinics.

There was no de facto cooperation between the CMs and the GPs because it turned out too difficult to reach the GPs during daily routine from the hospital. For this reason, the structured cooperation between CMs and GPs during the intervention was dropped.

Among other things, however, a systematic integration of general practitioners within the intervention was foregone. Only selectively should the general practitioners be addressed, within the study during the intervention. (Project manager, t3)

It was also found that the IMS and tablets needed technical modifications.

This preliminary study that we did didn’t go so well either, where we tested it for three months and in the end, it turned out that things that weren’t so good there were also confirmed afterwards and yes, that sometimes made the work not so nice. (Care manager, t3)

#### Implementation phase in the hospital: look for gaps in the daily routine to take action as CM

To implement the intervention, the CM would need to look for gaps in their daily routine in hospitals and act accordingly, even though they were employed by the respective hospital. As one of their professional duties, they identified suitable patients in the hospital information system of the respective site. They realized that they needed more patient-specific information for the interaction with the study patients and their relatives, such as “*How is the patient integrated into the family, or do they have any family at all, or things like that…*” (Care manager, t3). First, an attempt was made to establish direct contact with the potential participants and their relatives in the patient’s room. If this direct contact was not possible, hospital staff were asked to inquire about feasible times for a visit. CMs were then able to plan a visit with the potential study participants. In this context, the fact that the hospital staff were busy with their own ward duties made it challenging for them to provide additional support to the CMs. The study revealed that there was a lack of coordination between the CMs and other hospital staff. The CMs were not really integrated into the hospital staff structure. This particularly affected the flow of information between the groups.

(...) I have little contact with the medical staff because they are really extremely understaffed, also working a lot, so, I always see their work in the charts and in all the / CIS or whatever, where I can read everything, just like with the occupational therapists and physiotherapists / you encounter nurses in the hallway (...) (Care manager, t3)

This may have contributed to the lack of awareness of the project in hospitals, as indicated by the survey data (T2). Most hospital staff respondents could not rate their satisfaction with the project because they did not work on it directly. Of the 177 survey respondents (patients, relatives, GPs, hospital physicians, clinical nurses, and hospital social workers), 82 healthcare professionals stated that they were not aware of the project (see Fig. 4).

If the conversations about the study participation or about the study-specific content were successful and the patients had given their verbal consent to participate in the study, the CMs had to plan and design the further procedure independently. Patients underwent screening and baseline assessments to ensure they met the inclusion criteria for the RCT [3]. Subsequent to this phase of the procedure, the patients provided written consent and were randomized to the intervention and the control group, respectively. During this time, the CMs conducted comprehensive data collection from the patients and their relatives, primarily utilizing the iCM. However the creation of individualized treatment plans was partially possible due to outdated equipment and software:

So, we did certain assessments in the hospital and we also had this IMS that we had to orientate ourselves to, and actually only to this IMS officially. (Care manager, t3)

However, since further contacts with patients and relatives were not often possible, they were mainly treated at home: “*When the first interviews were practically carried out, we were then in the home, we carried out assessments there again and then the IMS was practically finished*” (Care manager, t3).

#### Outpatient care: emotional and mental support from CMs

**Fig. 2:**
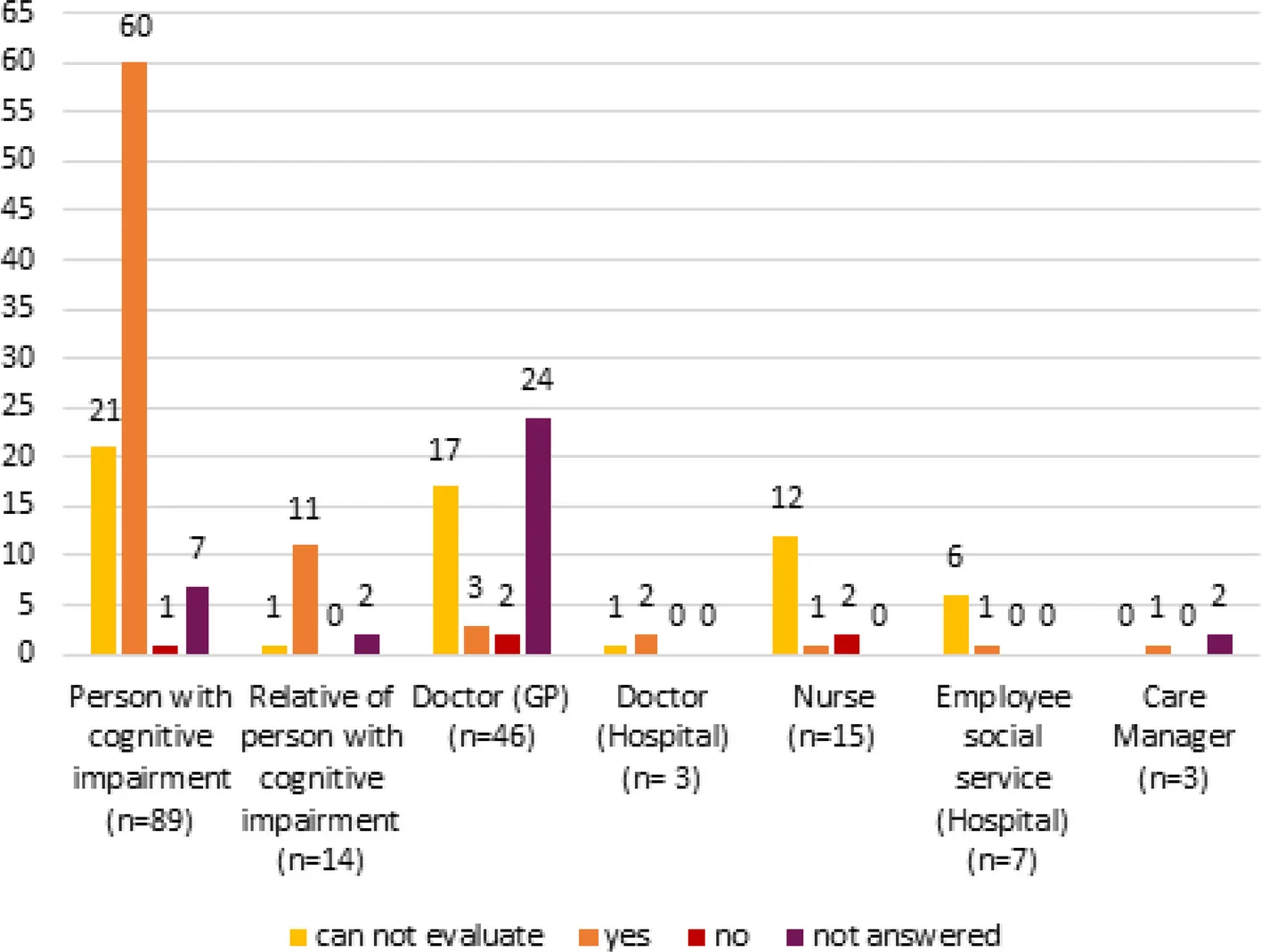
Satisfaction with the project (79 from total 177)

**Fig. 3:**
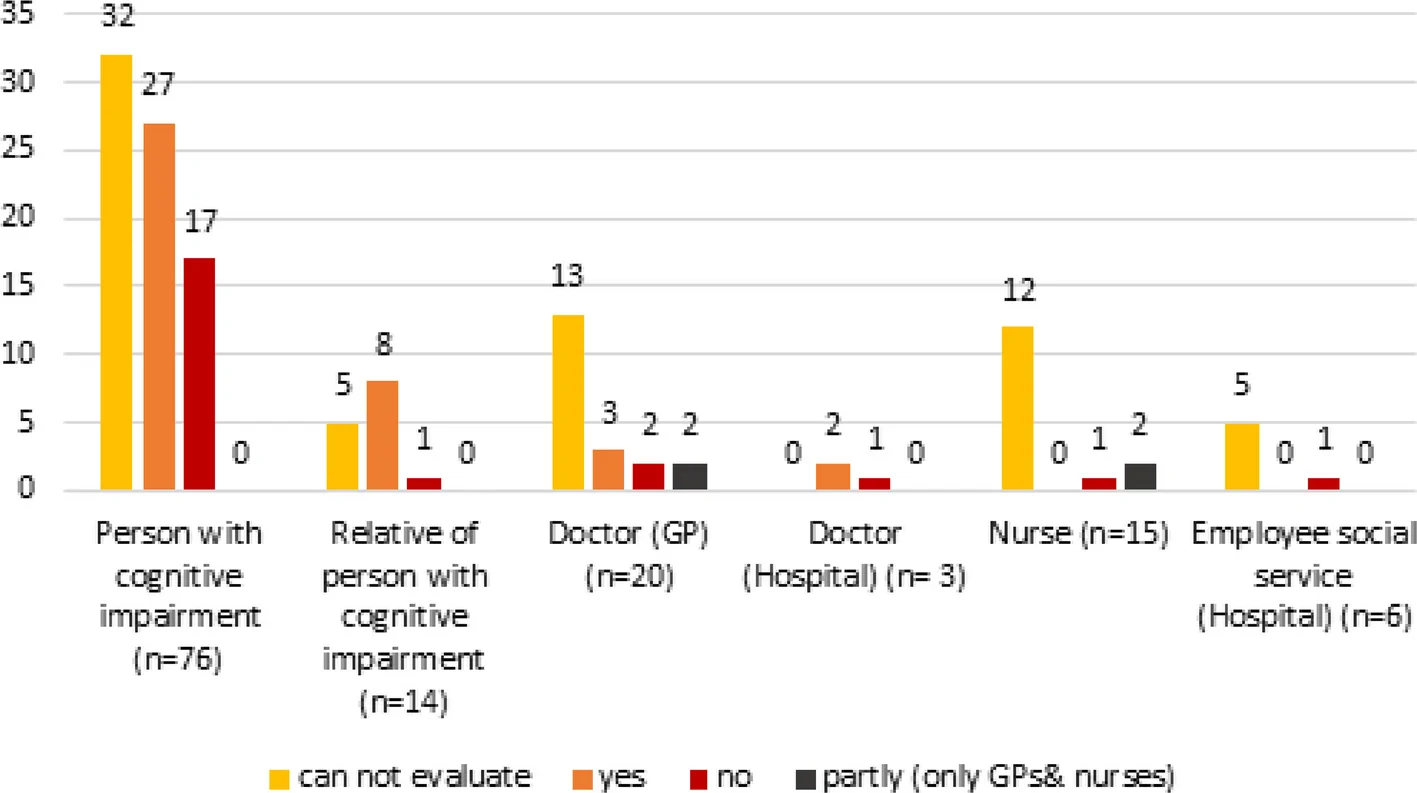
Facilitation of life/work by the CM

**Fig. 4:**
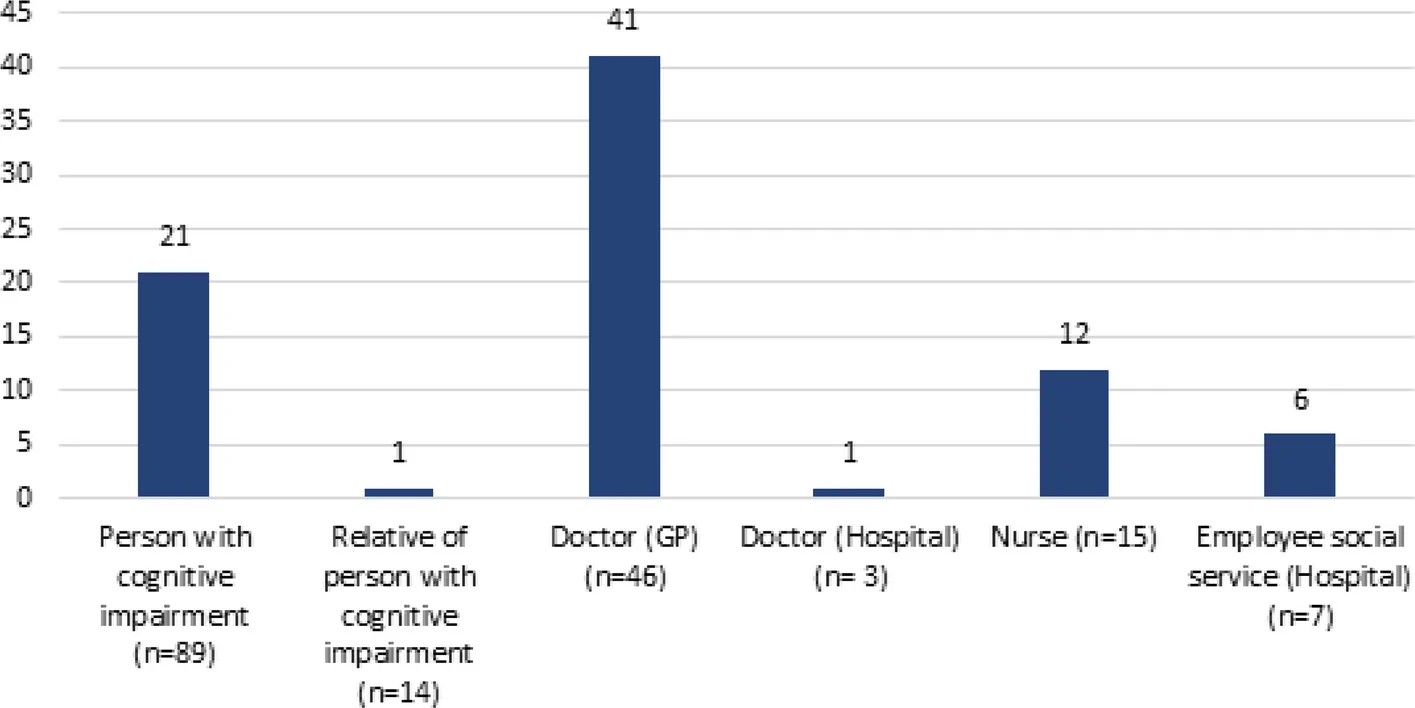
Project not noticed

**Fig. 5:**
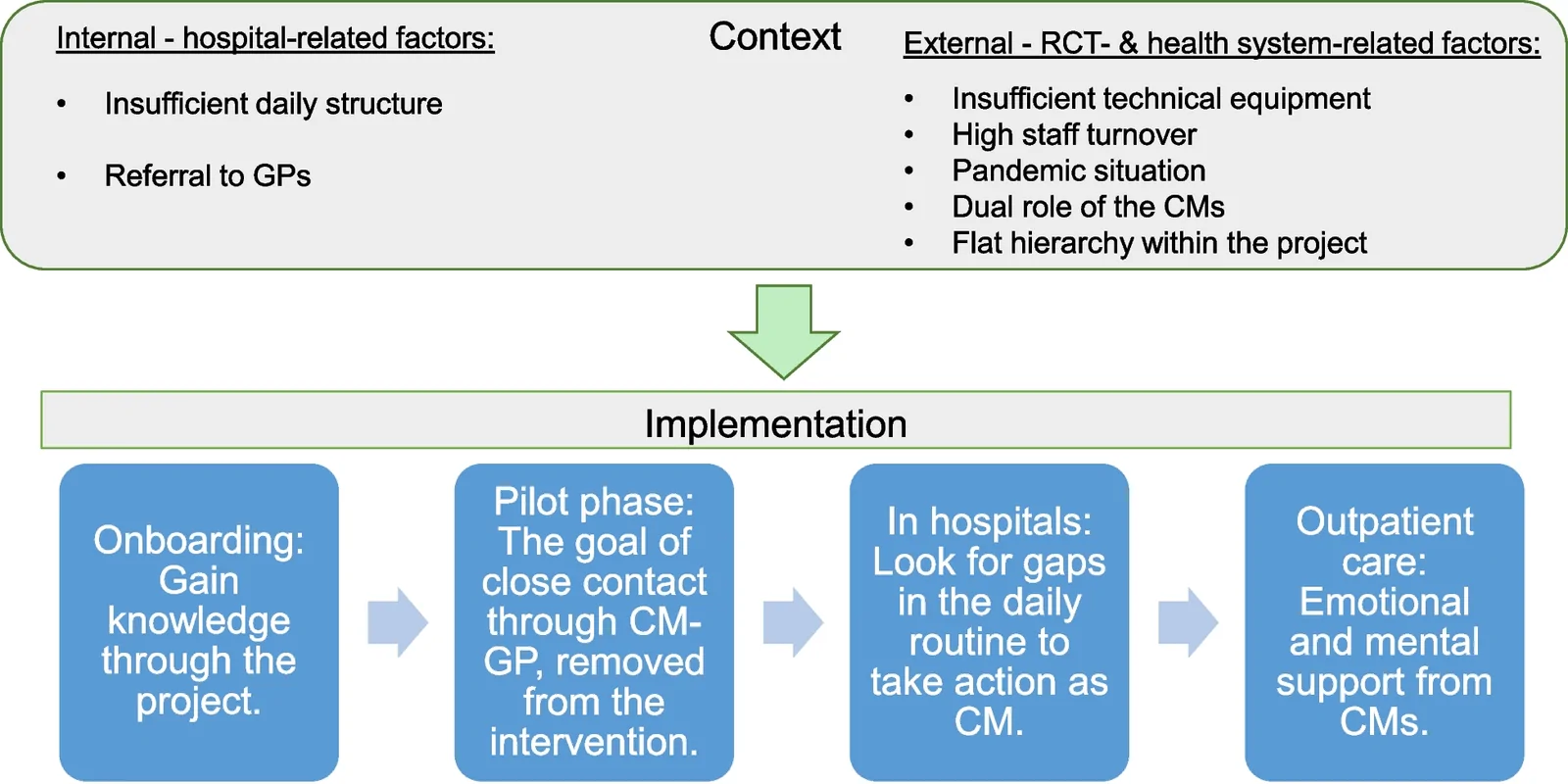
Overview of results from contextual factors to implementation outcomes

The quantitative data collection at time t2 (see Figs. 2, 3, and 4) reinforced the indications that the Care managers (CMs) interacted with the patients and their relatives (see Fig. 2) and an increasing number of patients as well as relatives saw a benefit of the intervention for their lives (see Fig. 3). These findings were confirmed and further explained by the t3 data from patients and relatives (Fig. 5).

After discharge from hospital, the patients and their relatives were visited by the CMs for further follow-up data collection as part of the RCT or in additional visits if they indicated a need for additional visits. If that was the case, the CMs in the home “*reacted to it somehow*” (Care manager, t3). Needs in this case often include emotional and mental support regarding the challenging situation: “*… I really had a lot of pastoral conversations, that has to be said quite clearly, yes*” (Care manager, t3).

Interviewees reported very little cooperation between the CMs and the GPs. Close contact with this group was dropped as a target in the RCT after the pilot phase. However, contact with the GPs was still made when the patient or relatives needed it. Therefore, the CMs stated that they had not coordinated their activity with the GPs on further outpatient care because they did not see this as their role:

... that somehow contact should be made with other specialists or something, I didn’t suggest that much at all because it wasn’t suggested to me. (Care manager, t3)

In addition, there was no feedback to the hospital about the continuation and success of the measures initiated there.

From the perspective of relatives of patients with cognitive impairments, the contact with the CMs was described as supportive. Through their visits, provision of information, and care, the CMs had given the affected patients/relatives a sense of security in a situation that was unfamiliar to them. The needs of the patients and their relatives were addressed, and open questions were addressed. For most patients, the contact with the CMs was a welcome change in their daily life. The personal exchange, in which also private matters could be discussed, led to the wish to have the outpatient visits take place more often:

Yes, she could have come more often, that was a nice conversation. Yes, it was very nice, I must say. (Patient, t3)

### What is delivered

It was possible to implement the iCM intervention in the clinics in Bielefeld and Greifswald, but the delivery intervention has produced the changes.

The assessments within the RCT (baseline and T1) in the hospitals, 3 months after hospital discharge (T2), and 12 months after hospital discharge (T3) were understood as the initial tasks by the CMs within the RCT. The majority of this was carried out as planned and the data collection times could mostly be adhered to.

Assessing needs, developing individualized treatment plans, and coordinating and monitoring needs were in many cases difficult to implement due to a variety of contextual factors in hospitals mentioned above. Nevertheless, the cognitively impaired patients and their relatives were largely satisfied with the CMs activities, although home contacts could mainly only be carried out at the times specified for assessment in the RCT.

Because of the timed routines in the clinics, the gaps between the different sectors and the difficult interaction between the different healthcare professions, such as physicians, nurses, and therapists, could not be overcome and coordinated as planned for in the project.

It was difficult to achieve a homogeneous level of knowledge within the project as not all CMs in the study received the same training due to the high turnover within the project. However, the high level of qualification and practical experience in patient care that some of the CMs brought with them was advantageous at this point.

Similarly, most healthcare professionals were unable to evaluate their satisfaction with the project in the T2 survey due to high staff turnover in hospitals and a lack of direct project involvement (see Fig. 3). This finding was further supported by t3 qualitative data, which indicated that many hospital staff had only minimal contact with the intervention in their daily routines and therefore felt unable to assess it.

Triangulation of t2 and t3 data suggests that, within hospital settings, CMs were required to operate within the constraints of existing workflows. As a result, their capacity to influence information exchange between teams, modify on-unit processes, or actively shape discharge planning remained limited. Qualitative insights from t3 interviews further highlighted structural and organizational barriers that restricted opportunities to streamline communication, coordinate care across units, and optimize the discharge process.

In contrast, both t2 and t3 data consistently demonstrate that, in the ambulatory setting, CMs were able to establish more continuous and meaningful contact with patients and their relatives. While t2 survey results reflected high levels of satisfaction with CM support, t3 interviews provided deeper insight into how this support was enacted. CMs described tailoring their activities to individual needs, accompanying patients to medical appointments when required, supporting families in navigating the healthcare system, and facilitating access to additional healthcare services.

## Discussion

The results of the present process evaluation show the practical implementation of the iCM intervention as a complex intervention with several interacting components. The implementation was carried out at different organizational levels of healthcare and resulted in improved patient/relative orientation. The CMs were only partially successful in assessing needs, developing individual treatment plans, and coordinating and monitoring the needs of people with cognitive impairments and their relatives. Collaboration with the different actors of the healthcare system was difficult for the CMs. These results highlight areas with potential for improvement and are thus important for the implementation of intersectoral care for patients with cognitive impairments and support for their relatives. Such areas for improvement include (a) the cooperation of CMs with the professionals involved in care, (b) the coordination of hospital discharge planning with the GPs, and (c) the monitoring of recommendations from the hospital in the primary care setting and in the patients’ homes.

Several publications include views on what constitutes collaboration in an intervention setting and formulate intended actions in the process of preparation as well as during the implementation of a complex intervention [20, 24, 39]. In this study, the iCM intervention originally developed for the outpatient setting as part of the previous DelpHi-MV study [3, 40, 41] was modified and extended to the management of patients with cognitive impairments after discharge from an inpatient treatment in the hospital. This transsectoral transition was the focus of the intersec-CM study. Of crucial importance in the process evaluation were the predefined qualifications of the CMs and the implementers and their training, the type of discharge management in the respective hospital as well as the consideration of the care reality in German hospitals (e.g., high staff turnover) [42–44]. The CMs were trained on topics such as the general project-specific content of the intersec-CM RCT, geriatric psychiatry, and the delirium prevention program in hospitals. However, the training measures for the CMs, which are based on a specially developed concept [19, 20, 27], as well as continuous monitoring through case conferences [45] were missing in the implementation process. This means that a significant proportion of CMs’ responsibilities should involve working closely with everyone involved in the inpatient setting. For future implementation of the iCM intervention, training should be adapted to cover specific topics, such as effective communication and collaboration with hospital doctors, nurses, social workers, and GPs.

Additionally, the hospital context should be examined in advance, and specific training content should be developed and integrated into CM training, taking into account the CMs’ qualifications and the specific discharge management procedures at each hospital, because an adaptation of the implementation to the setting and the consideration of the needs of the involved actors [21, 27, 46–48] could have simplified the implementation of the complex iCM intervention in the already complex setting (double complexity) [49, 50].

The monitoring of hospital recommendations should be supported by the computer-based IMS, which in this study was technically insufficient and not equipped with up-to-date programming. The previous DelpHi-MV study demonstrated that a well-functioning IMS allows systematic identification of unmet needs and improves monitoring of the success of treatment and care plans [15, 40, 41]. Therefore, the technical condition of the IMS should be carefully considered in future implementations to ensure its effective use, as it could facilitate the CMs’ work in carrying out the “monitoring of hospital recommendations” component of the intervention.

In conclusion, it should be noted that, although the intervention could be implemented to some extent, the need for iCM was clearly reflected in the qualitative interviews with experts, patients’ relatives, and GPs who participated only partially in the study (t3). GPs reported that iCM could improve the flow of information and potentially save resources, while hospital staff indicated that iCM could optimize interface management and enhance case-related care. This benefit is supported by the literature, which recommends practicing such interventions [2, 3, 51, 52]. However, it is evident that implementation should be guided by prescribed theoretical frameworks. Careful, theory-based development of complex interventions is essential to ensure that their limitations in application and evaluation are properly addressed. Furthermore, the development of the iCM intervention should involve a wide range of stakeholders—including scientists, patients and their relatives, physicians, nurses, social workers from hospitals, GPs, specialists, physiotherapists, and outpatient nurses—to enhance the feasibility and practicability of the intervention [46].

### Limitations

It is important to note that this process-evaluation-study has some limitations.

One of these limitations relates to the timing of the interviews and survey data collection. The intervention began in the clinics on 1 November 2018, with T1 interviews conducted in December 2018 and subsequently between March and June 2019. As implementation and interviews coincided, careful consideration was given to selecting interview participants who were not involved in the project, to minimize any potential impact on their perceptions of routine care. However, it should be noted that respondents were made aware of the study content in the clinics, and it cannot be ruled out that this influenced their responses.

In addition, it was not possible to conduct the t2 survey until the summer of 2020. By that time, the intervention had been implemented in the clinics for a year. This delay in conducting the survey may have affected the results, given the high turnover of healthcare professionals in Germany. It is therefore likely that a considerable fraction of the clinicians and other staff were not aware of the project. This could undermine the validity of the survey results.

The next limitation relates to the fact that only respondents who volunteered for the study were interviewed. A selection bias can be assumed here, which is also confirmed by the low initial response rate and the necessary reminders. Therefore, it can be assumed that only people who were interested in the topic and willing to talk about it shared their insights. This might result in an absence of critical stakeholders. But critical feedback may also have led someone to want to participate in the research at all costs. However, we believe that this enabled us to receive both positive and critical feedback and that the process was well represented, also thanks to the supplementary work. Overall, it should be noted that due to staff turnover, not all iCMs received the same training. This inconsistency affects the accuracy of the intervention and comparability between sites. This could have impacted the validity, results, and generalizability of the study.

Originally, there was a much closer link between the activities of the DeCM in clinical and general practice settings. In fact, the pilot phase showed that the logistical effort involved was too high and interest among practices was too low. As a result, the DeCM maintained contact with patients after discharge but only established contact with practices on an ad hoc basis and when necessary.

Furthermore, it must be taken into account that the results of the study do not reveal any clear mechanisms showing how iCM influences the results. This means that the underlying mechanisms are only partially understood.

### Conclusions

The present process-evaluation-study could again underline the necessity and meaningfulness of process evaluations to understand the functionality and procedural feasibility of an RCT. Through the study, significant insights into the “black box” of the implementation of the iCM intervention could be gained. Notably, concrete implications for research and practice should be that, for the successful implementation of such a complex intervention as iCM, it is necessary to apply evidence-based constructs step by step. It is of crucial importance to conduct a more in-depth analysis of each hospital’s individual setting prior to the start of the implementation, as well as considering the qualifications of the CMs and their training, the nature of discharge management in the respective hospital, and the care reality in hospitals, which is characterized by high staff turnover.

Including the project management and scientific staff of the project sites in Bielefeld and Greifswald as interview partners in the t3 qualitative interviews survey enabled a comprehensive understanding of the project’s processes and the implementation of the intervention. In the future, the project management and scientific staff of the project should be included in the t1 interview of the process evaluation earlier, ideally immediately after the pilot phase of the RCT. This would provide an opportunity to sensitize them during the carrying out the RCT and to make them aware of possible deviations within the intervention. Process evaluation can thus contribute to a reflective approach within the RCT. So, this open exchange between RCT staff and process evaluation staff is crucial for this.

## Supplementary Information


Supplementary Material 1.Supplementary Material 2.Supplementary Material 3.Supplementary Material 4.

## Data Availability

The datasets created and analyzed during the current study are available from the corresponding author on reasonable request.

## Abbreviations

ANT: Actor-Network Theory
CMs: Care managers
DeCM: Dementia care management
DelpHi-MV: Dementia: Life- and person-centred help in Mecklenburg-Western Pomerania, Germany
GPs: General practitioners
MRC framework: Guidelines of the British Medical Research Council for the implementation and process evaluation of complex interventions
IMS: Information management system
iCM: Intersectoral care management
DFG: Memorandum for the Preservation of Good Scientific Practice
RCT: Randomized controlled trial
