# Supplementary material for: Process evaluation of the randomized trial—“Care management for older people with cognitive impairment during and after hospitalisation in Germany (intersec-CM)”
Source: Trials. 2026 Aug 29;27:553. doi: 10.1186/s13063-026-09965-0 (PMC13525668; doi:10.1186/s13063-026-09965-0)
Supplement: Supplementary file 2 [file 13063_2026_9965_MOESM1_ESM.docx]

## Categories -, code scheme, exemplary quotations, Coding Rule

|  | Category and Code Label | Definition | Exemplary quotations | Coding Rule |
| --- | --- | --- | --- | --- |
| 1 | **Context** | Conditions in the hospitals that could have an influence on the implementation of the intervention | - | - |
| 1-1 | **Internal - hospital-related factors** | Conditions and processes that are specific to the hospitals. | - | - |
| 1-1-1  1-1-1-1 | **Insufficient daily structure**  **Rigid and inflexible structure** | Daily routine–specific processes within the hospitals | "... to always have to interfere in this everyday life and to meander through in order to really come into contact with the patients and also to be able to conduct an assessment, that was always very challenging" (CM t3) | Interview content referring to daily clinical routines |
| 1-1-2 | **Referral to GPs** | Transition processes from the hospital to GPs | "So maybe the problems are the GPs. I think so because our doctors don't communicate with the GPs like that. We can't get close to the GPs, who are sometimes unwilling to do anything.” (Social worker, t1)  „Not at all, we didn't (did we?) collaborate with the GPs.“ (CM, t3) | Statements by interviewees regarding collaboration with general practitioners during patient discharge from hospital |
| 1-2 | **External - RCT- and health system-related factors** | Conditions and processes extending beyond the project or related to the health system framework | - | Statements referring to project-specific conditions or system-related aspects that were linked to causal relationships with the intervention |
| 1-2-1 | **Insufficient technical equipment** | Technical problems with tablets or IMS software | "(...) yes, so for me the biggest problem was that this IMS is still not so mature, that there, so that it can never be perfect, but that it, that it was further worked on or that it was intensified, that was already very (...) almost said larifari, (...)" (CM, t3) | Statements regarding problems with the technical equipment required to implement the intervention |
| 1-2-2 | **High staff turnover** | Staff turnover within hospitals or within the project | “So this is (laughs) one of my projects where the staff turnover was the biggest (laughs) so it was quite bad, staff turnover, in the planning of the project there were others who were in the implementation and then again others who are now in the further implementation, a huge problem." (Project management, t3)  "... we also had extreme staff turnover in the hospital, so that contacts simply kept changing like crazy, and in some cases the processes for their routine work weren't even clearly defined..." (Physician, t3) | Statements referring to new staff members in hospitals or the project team, or explicitly addressing staff turnover. |
| 1-2-3 | **Pandemic situation** | Specific conditions during the COVID-19 pandemic | "(...) because of the coronavirus, the home visits were cancelled afterwards, and it is simply difficult to cover everything in a telephone survey.” (CM t3) | Statements regarding pandemic-related measures that influenced the implementation of the intervention |
| 1-2-4 | **The dual role of the CMs** | Responsibilities of the CMs within the project | "This is a very difficult role because they have this double role. On the one hand, they are a case manager or care manager and are responsible for the immediate needs of the patients, and they also feel responsible. And on the other hand, they also have the role of a study assistant. And that is, I think, difficult, to change hats more often." (Project management, t3)  „And that's what I've been learning over the last few weeks: ‘OK, this is the scientific role now, and it's just as much your job as being the care manager.“ (CM, t3) | Statements referring to the responsibilities within the project that were to be carried out by CMs |
| 2 | **Implementation** | Processes related to the implementation of the intervention | - |  |
| 2-1 | **Onboarding: Gain knowledge through the project** | Training and dissemination of information about the project within hospitals and internally within the project | "We met once and looked at some assessment instruments for half a day." (Nurse, Project management in hospital, t3)  “Public relations within our hospital, occasionally posting updated articles about the project on our website or featuring the project in staff magazines, where it’s presented again. And similar activities.“ (Nurse, t3) | Statements regarding training activities and information dissemination within hospitals |
| 2-2 | **Pilot phase/adaptation of the intervention: The goal of close contact through CM-GP, removed from the intervention** | Implementation of the pilot project | "This preliminary study that we did didn't go so well either, where we tested it for three months and in the end, it turned out that things that weren't so good there were also confirmed afterwards and yes, that sometimes made the work not so nice." (CM, t3)  „Among other things, however, a systematic integration of general practitioners within the intervention was foregone. Only selectively should the general practitioners be addressed, within the study during the intervention.“ (Project management, t3) | Statements regarding the pilot phase of the project and its consequences |
| 2-3 | **In hospitals: Look for gaps in the daily routine to take action as CM** | Collaboration with hospital staff; gathering patient information; development of individual patient plans | „If I still had questions that I couldn't find answers to in the hospital information system, I would ask, or if I had the impression that something had happened to the patients, I would usually follow up with the nursing staff. It was a collegial exchange, but it was less support from me to the nursing staff and more the other way around (laughing), with them giving me additional information about the patients.“ (CM, t3) | Statements by CMs and hospital staff regarding actions related to the project, communication, and collaboration processes |
| 2-4 | **Outpatient care: Emotional and mental support from CMs** | Care of patients and relatives in the outpatient setting | “... I really had a lot of pastoral conversations, that has to be said quite clearly, yes." (CM, t3 )  "Yes, she could have come more often, that was a nice conversation. Yes, it was very nice, I must say." (Patient, t3)  “And I also found it very important that she said again to consult neurologists again.” (Relative, t3)  “So, I thought it was really great that we moved in there as a family.” (Relative, t3) | Statements by CMs, patients, and relatives regarding actions at patients’ homes or communication between them |

Abbreviations: CM: care manager, GP: general practitioner, IMS: computer-based information management system, RCT: randomized controlled trial
