# Supplementary material for: Process evaluation of the randomized trial—“Care management for older people with cognitive impairment during and after hospitalisation in Germany (intersec-CM)”
Source: Trials. 2026 Aug 29;27:553. doi: 10.1186/s13063-026-09965-0 (PMC13525668; doi:10.1186/s13063-026-09965-0)
Supplement: Supplementary file 4 [file 13063_2026_9965_MOESM4_ESM.docx]

**TRLS-D-25-00210**

**Process evaluation of the randomized trial – “Care management for older people with cognitive impairment during and after hospitalisation in Germany (intersec-CM)”**

Chikhradze N, Hannich A, Otte I, Busse TS, Suslow A, Nikelski A, Schumacher-Schoenert F, Kreisel SH, Boekholt M, Thyrian JR, Hoffmann W, Vollmar HC.

Dear Naoki Kodama, dear reviewers,

We are grateful for the thorough review process; the comments were very helpful and increased the quality of our paper.

Below you will find a point-to-point list of replies to the reviewers’ concerns. Substantial changes in the manuscript are marked in yellow color.

We hope that the revised version of the manuscript will now be suitable for publication.

Thank you for your time and consideration.

Yours sincerely,

Nino Chikhradze for the authors

|  | **Reviewer #2** |  |
| --- | --- | --- |
| 1. | Thank you very much for the careful and substantial revisions. The manuscript has clearly improved, especially with regard to the background and the formulation of the research questions.  Some issues, however, still require further refinements. As outlined in my detailed comments, these are the consistent use of terminology and concepts across the manuscript, the accuracy of referenced study protocols, and the clearer structuring of results in relation to the research questions. In addition, the current presentation of "mechanisms of impact" still reflects implementation factors rather than explanatory pathways as defined in the MRC guidance. | We would like to thank you very much for your helpful feedback. We have implemented your suggestions, which have greatly improved our manuscript. |
|  | Background |  |
| 2. | The overall aim of the process evaluation and the research questions are now much clearer. To further strengthen alignment with the MRC framework, it may help to indicate for each research question which MRC dimension it reflects (context, implementation, or mechanisms of change). | We incorporated your feedback and linked each question to its corresponding MRC dimension. P. 6 L: 138 - 143 |
|  | Methods |  |
| 3. | Page 7, lines 158 ff.  Thank you for addressing my earlier comments regarding the use of the MRC framework and for revising this section.  However, the description of the aims of the three data‑collection phases (t1-t3) still does not align clearly with the four research questions derived from the MRC framework.  The focus on contextual factors at t1 and on acceptance and implementation at t2 and t3 only partially reflects the research questions on contextual conditions, implementation steps, delivery/intervention changes, and mechanisms of impact. In particular, "mechanisms of impact" are not represented in the current description, and "acceptance" is not part of the stated research questions. The wording used in Figure 1 is also not consistent with the research questions or with this description. I recommend using consistent wording and concepts throughout the manuscript.  In addition, the manuscript introduces the three data‑collection time points relatively late, without indicating earlier that the process evaluation was conducted across multiple phases. This makes the study design and logic more difficult to follow. | Thank you for your feedback. We have implemented all the suggested changes across the manuscript and appreciate your detailed notes. P. 7 – 8; L: 158  Specifically, we:  Aligned the description of the three data collection phases (t1–t3) with the four research questions derived from the MRC, ensuring consistency in wording and concepts throughout the Methodes.  We have specified the t1, t2, and t3 objectives and hope this will enhance clarity.  Incorporated mechanisms of impact and clarified the role of acceptance within the context of t2 and t3.  Updated Figure 1 wording to reflect consistent terminology aligned with the research questions. P. 11; L 264 - 281  Emphasized earlier that the process evaluation spans multiple phases, clarifying the study design and logic up front to improve readability. P. 7 -8 L: 159 ff. |
| 4. | Page 7, Lines 167 f.  The manuscript states that the procedures of the process evaluation were defined in a study protocol; however, the cited reference does not describe the procedures of the process evaluation but rather those of the RCT. My understanding is that the protocol for the process evaluation was published elsewhere. I recommend checking the citation and correcting the reference accordingly. | We have corrected this citation; thank you very much for the comment. P.7 L. 183 |
| 5. | Page 17, line 401  See my previous comment regarding the use and application of these concepts. | We have changed this sentence. Thank you very much for the comment. P. 19 L. 450 - 451 |
| 6. | Page 22, lines 537 ff.  Thank you for revising the section on "mechanisms of impact".  However, the revised text still mainly describes facilitating and hindering factors for implementation. These are important findings, but they do not yet correspond to what the MRC process evaluation guidance defines as "mechanisms of impact," namely the underlying processes that explain how and why the intervention (and its single components) leads to certain outcomes. These explanatory pathways are not yet clearly articulated or linked to a programme theory or logic model.  If the data do not allow for a more theory‑driven identification of mechanisms, it may be worth reconsidering whether this dimension should be reported. Alternatively, the authors could clarify how the presented findings are intended to represent mechanisms within the study | We agree with you and have integrated this part into Chapter 3.4 “What is delivered.” We have removed the title 3.5 because the available data do not allow us for such program-theory development. Consequently, we are omitting this dimension and focusing instead on Context and Implementation in this publication. |
| 7. | Other comments:  Thank you for your response. I would like to clarify that my original comment concerned the reporting guideline you used, not the conceptual framework guiding the process evaluation.  In my initial review, I noted that applying a reporting guideline for RCTs (CONSORT) is not appropriate for reporting a mixed‑methods process evaluation. In your reply, you refer to the MRC Process Evaluation Guidance. I fully agree that the MRC guidance is the correct framework for conducting a process evaluation. My point, however, was about the reporting guidline, not the conceptual framework.  Reporting checklists such as COREQ, SRQR, or STaRI are better suited for reporting qualitative or mixed‑methods process evaluations and would more accurately reflect the methodological components of your study.  I hope this clarifies the intent of my initial comment. | Thank you for this feedback. The COREQ Checklist has been added as an appendix. P. 7; L. 161-162 |

|  | Reviewer #3 | We are very grateful for your valuable feedback and recommendations. We have made efforts to incorporate your suggestions into the manuscript, and your feedback has significantly improved its content. |
| --- | --- | --- |
| 1. | I was not one of the earlier reviewers of this manuscript. It has potential to be a useful manuscript, but lack of coherence and alignment with behaviour change theory, combined with the age of the data, mean that it currently does not offer a novel contribution to the literature. | Thank you very much for this valuable feedback.  Within this process evaluation, we identified both facilitating and hindering aspects of the implementation. These can be found in the chapter on contextual factors. Since this publication focuses on the implementation steps, these factors are not described in detail.  Unfortunately, we did not use behavior change theory for the data analysis. |
|  | Abstract |  |
| 2. | Abstract: "Successful" is a strange term for describing care, could you consider an alternative phrase?  The care model is not described in sufficient detail within the abstract for the reader to understand what has been implemented. | We changed this phrase to “effective care” and described the iCM model in the abstract. P. 2; L: 28-37 |
|  | Background |  |
| 3. | Please refer to terminology on how to refer to people with cognitive impairment/dementia - a "special vulnerable group" is not person-centred or in line with the preferences of people living with dementia.  What do the authors mean by "special tasks", this is unclear. | Thank you for your feedback. We have corrected the error and removed the word 'special'. P. 4; L: 76 |
| 4. | Methods |  |
| 5. | I was surprised to see the authors had not aligned their process evaluation with a model of behaviour change, for example to examine barriers and facilitators to implementation, or to explore what would support normalising this intervention in practice. This feels like an oversight and reduces the usefulness of the findings in clinical care.  The rationale for recruiting individuals who weren't involved in the RCT, as part of the process evaluation, should be provided. | Please see our respond on your first comment about barriers and facilitators to implementation.  Within this process evaluation, we developed practical implications; however, they are not presented in this publication, as the manuscript focusses on the implementation.  We have added this content and hope that the reasons for including the additional interview partners – please see our respond on your comment number 6. |
| 6. | The rationale for recruiting individuals who weren't involved in the RCT, as part of the process evaluation, should be provided. | We have added this content and hope that the reasons for including the additional interview partners in this process evaluation are now presented more clearly. P. 8 L. 196 - 203 |
| 7. | Please report details of the qualitative sections of the process evaluation in line with COREQ guidance. | Thank you for this feedback. The COREQ Checklist has been added as an appendix. P. 7; L. 161-162 |
| 8. | More detail is needed about the analysis conducted. What form of qualitative analysis was conducted and why was this chosen? Without knowing this the reader cannot be sure that appropriate steps of analysis have been followed. | We have added this content. Thank you very much for your comment. P. 10; L. 239 - 241 |
|  | Results |  |
| 9. | The results should open with an overview of the structure of the themes (or alternative phrasing). | The structure of the themes was introduced at the beginning of the results chapter. P. 12; L. 294 - 299 |
| 10. | The authors refer to chapters 4.2, 4.3 and 4.4 - what are these, and why are they described in this way? | The numbering of the chapters has been corrected, and we hope this has improved clarity. P. 12; L. 294 - 299 |
| 11. | For the survey, please include the % completion rate than n=177 equals to. | We have added the correct percentage. P. 13; L. 320 |
| 12. | When reading the qualitative results, the opportunity to map to a behavioural science framework is a missed opportunity. Thought should be given to ensuring that within the results, the unique factors associated with this intervention, rather than healthcare service delivery in general, should be highlighted and drawn out.  An example of where this is done well is the section on high staff turnover, where the authors could expand on this to provide more detailed quotes about why staff turnover caused such significant issues for implementation. | We have incorporated your recommendation and updated the results accordingly. We have expanded the section on high staff turnover and provided more detailed quotes about why this caused significant issues for implementation.  We hope that these revisions effectively address the issues that were causing significant challenges to implementation. P. 16; L. 385 – 396. And P. 17; L 404 -411 |
| 13. | The triangulation of data felt lacking and the authors could more clearly draw this out. | For more clarity, we have added sections on data triangulation both in the Methods and Results sections. We hope that these additions have contributed to improved clarity.  P. 11, L. 258-291; and P.22, L. 535-538; and P.23 - 24 L. 585 - 600 |
|  | Discussion |  |
| 14. | The discussion is long and difficult to follow. It is hard for the reader to know exactly what the main points they should be taking away are. | We have condensed and refined the discussion to improve clarity and readability. We hope that these revisions make the key points easier to follow. P. 24 - 26 |
| 15. | Much of the data was collected over 6 years ago. How can the authors be sure that the findings presented here are still relevant? | We acknowledge that the data was collected 6 years ago; however, the findings remain relevant because they highlight enduring systemic issues—such as staff turnover, care coordination challenges, and dual roles of care managers—that continue to affect implementation of intervention in hospital-to-home transitions. Triangulation of quantitative and qualitative data further strengthens the applicability of these insights to current and future interventions. |
| 16. | A clinical implications section is lacking, meaning that the impact of the process evaluation on future care cannot be established. | We structured the discussion so that the implications for future implementation of iCM are embedded throughout the different sections of the discussion. P. 24 - 26 |
